# Association of loneliness with all-cause mortality: A meta-analysis

**DOI:** 10.1371/journal.pone.0190033

**Published:** 2018-01-04

**Authors:** Laura Alejandra Rico-Uribe, Francisco Félix Caballero, Natalia Martín-María, María Cabello, José Luis Ayuso-Mateos, Marta Miret

**Affiliations:** 1 Department of Psychiatry, Universidad Autónoma de Madrid, Madrid, Spain; 2 Instituto de Salud Carlos III, Centro de Investigación Biomédica en Red de Salud Mental, CIBERSAM, Madrid, Spain; 3 Department of Psychiatry, Hospital Universitario de La Princesa, Instituto de Investigación Sanitaria Princesa (IIS-Princesa), Madrid, Spain; Jerusalem Institute of Aging Research, ISRAEL

## Abstract

**Introduction:**

Loneliness has social and health implications. The aim of this article is to evaluate the association of loneliness with all-cause mortality.

**Methods:**

Pubmed, PsycINFO, CINAHL and Scopus databases were searched through June 2016 for published articles that measured loneliness and mortality. The main characteristics and the effect size values of each article were extracted. Moreover, an evaluation of the quality of the articles included was also carried out. A meta-analysis was performed firstly with all the included articles and secondly separating by gender, using a random effects model.

**Results:**

A total of 35 articles involving 77220 participants were included in the systematic review. Loneliness is a risk factor for all-cause mortality [pooled HR = 1.22, 95% CI = (1.10, 1.35), *p* < 0.001] for both genders together, and for women [pooled HR = 1.26, 95% CI = (1.07, 1.48); *p* = 0.005] and men [pooled HR = 1.44; 95% CI = (1.19, 1.76); *p* < 0.001] separately.

**Conclusions:**

Loneliness shows a harmful effect for all-cause mortality and this effect is slightly stronger in men than in women. Moreover, the impact of loneliness was independent from the quality evaluation of each article and the effect of depression.

## Introduction

Loneliness has been conceptualized as an individual’s subjective experience about the generalized lack of satisfying human relationships [1]. A recent article has shown that the prevalence of loneliness in European countries ranks from 10% in Western and Northern countries to 55% in Eastern countries [2]. Moreover, a report conducted in the United Kingdom suggested that if loneliness is not considered as a relevant priority, in 2030 depression and other health problems may increase, given their association with loneliness [3].

According to previous articles, loneliness has significant implications with several physical and mental health issues such as: depression [4, 5], alcoholism [6], cardiovascular problems [7], sleep difficulties [7], alteration in the immunological system [8], Alzheimer´s disease [9], and health status in general [10]. Moreover, an increasing body of research has shown that loneliness is also associated with early mortality [11–14].

In order to have a better understanding of the association of loneliness with mortality, gender analyses should be carried out for several reasons. Firstly, women live generally longer than men [15]. Secondly, some studies have shown that feelings of loneliness might be more prevalent in women than in men [16]. Thirdly, women and men build social networks in a different way, as an example, men experience smaller social networks [17] and less intimate relationships [18]. Fourthly, it is culturally less acceptable for men to express their emotions than it is for women [19]. And last but not least, some common risk factors for loneliness are also gender specific, i.e.; depression is more prevalent in women than men [20] whereas alcoholism is more frequent in men [21]. Moreover, the longer life expectancy of women entails that some risk factors for loneliness such as living alone and widowhood, occur earlier in women than in men [22].

There is a lack of research on the gendered aspects of the association of loneliness with all-cause mortality. To our knowledge, no meta-analysis that studies this association by gender has been carried out yet, and this is problematic because what could be associated with mortality for the whole sample might not be for men or women separately. One recent meta-analysis analyzed the impact of loneliness on mortality in men and women together [23]. This meta-analysis, though valuable, did not conduct sensitivity analyses for the quality of the studies, covered only a specific range of years and was limited to studies published in English. Therefore, the main aim of this meta-analysis is to determine whether loneliness is associated with all-cause mortality, considering all populations (including general and clinical populations). A secondary aim is to check whether this association is the same in women and men. Additionally, this meta-analysis has been conducted with no languages and time restrictions.

## Methods

Pubmed, PsycINFO, CINAHL and Scopus databases were searched for articles that measured loneliness and mortality published until June 27^th^, 2016. In these databases, all the abstracts of the articles are provided in English, even though the articles might be in other language. The following terms were used to search all articles in the databases: (("Loneliness"[Mesh]) OR Lone*[Title/Abstract]) OR Forlorn*[Title/Abstract]) OR Desol*[Title/Abstract]) OR ("Social Isolation"[Majr] OR "Feeling isolated"[Title/Abstract]) AND ("Mortality"[MESH] OR "Death"[Mesh] OR Decease*[Title/Abstract] OR Die[Title/Abstract] OR Dead[Title/Abstract] OR Remain alive[Title/Abstract] OR Remained alive[Title/Abstract] OR "Longevity"[Mesh] OR "Survival"[Mesh]) AND (Humans[Mesh]) AND (adult[MeSH]) NOT ("Cross-Sectional Studies"[Mesh]) NOT ("Books"[Mesh]) NOT ("Validation Studies" [Publication Type])). Search terms were tailored to each database. In addition, in order to minimize omissions, the reference sections of past reviews and meta-analysis were examined to locate articles not identified in the search.

The inclusion criteria were: articles with longitudinal observational design, prospective cohort design, meta-analysis, and systematic reviews. Articles that selected participants older than 18 years, and that used loneliness and mortality as measures of interest were included too. Articles in which mortality was the outcome measure, and loneliness was the independent variable defined as a subjective feeling that accompanies the perception that one’s social needs are not being met by the quantity or especially the quality of one’s social relationships, were also included.

The exclusion criteria were psychometric studies (development or validation of questionnaires or scales), articles of phase-I/II clinical trials, cross-sectional, primary prevention, ecologic, case report/case series, retrospective, and case-control studies. Non-human population, articles that did not analyze loneliness and mortality, articles that did not evaluate loneliness or perceived feelings of social isolation but other constructs such as size of the network, articles that did not consider loneliness as an independent variable, and articles that did not consider mortality as a dependent variable were also excluded. Since the aim of this study was to analyze the association of loneliness with mortality through physical disease, articles investigating death by suicide, injury, or accidents were not included. Thesis and books or book sections were excluded as well.

Three subsequent steps were performed to select the articles and collect the data. In the first step, articles with prospective and longitudinal design that addressed the effect of loneliness on mortality were identified and selected. A software package for managing bibliographies was used to eliminate duplicates. A researcher (LARU) checked the titles and abstracts of all the articles for inclusion or exclusion. In case the article was excluded the reason was provided. A random sample of 337 (20%) of the articles was double-checked independently by a second researcher (NMM). This 20% was selected with the statistical software SPSS. Initial disagreements between reviewers were solved by discussion; if no agreement could be reached a third researcher (MM) was consulted. In the second step, all included articles were fully read to confirm that they fulfilled all inclusion criteria. In the third step, objective and verifiable characteristics of each included article were extracted. In articles that presented more than one analysis, the one that adjusted by more confounders and the one that reported more causes of mortality was selected. When multiple effect sizes were reported across different levels of loneliness, the effect that was reported as “often lonely” or “severe/chronic loneliness” was extracted. Also, when effect sizes by different type of loneliness were reported, the emotional loneliness value was selected. Throughout this work the term “articles” will be used for papers found in the systematic review, while the word “studies” will be employed for papers included in the meta-analysis where the analysis and the effect sizes are provided separately for men and women or for different age groups. If there were doubts about the analyses or if the methodology was not clear, authors were contacted by e-mail.

The articles included in the meta-analysis were assessed for quality using The Cochrane Risk of Bias Assessment Tool for Non-Randomized Studies of Interventions (ACROBAT-NSRI) [24]. This tool includes seven domains: 1) Bias due to confounding, 2) Bias in selection of participants, 3) Bias in measurement of interventions, 4) Bias due to departures from intended interventions, 5) Bias due to missing data, 6) Bias in measurement of outcomes (in this case mortality), and 7) Bias in selection of the reported result. Since this meta-analysis did not include articles of interventions items three, four, and five were omitted. The item related to bias due to missing data was not considered because the dependent variable was mortality and the majority of the articles evaluated it with death registries, so they did not have missing data, and furthermore the potential bias related to the cause of death was already evaluated in item 6. Additionally, a new item that evaluated bias in the measurement of the independent variable (loneliness) was added. The response options for an overall judgment are: low risk of bias, moderate risk, serious risk, critical risk and no information. To consider an article with low risk of bias it is necessary to score in all items low risk. If at least one item was evaluated with moderate risk, the article was evaluated as presenting moderate risk of bias, the same if at least one item was considered as serious risk of bias. For bias due to confounding it was considered low risk if the article adjusted for age, sex, health status (considering chronic diseases as a possible indicator), socioeconomic status (considering education and occupation as proxy variables), smoking, and depression or anxiety; for bias in selection of participants, it was observed whether it counted with consecutive or random recruitment of participants or representative populations; for bias in measurement of mortality it was checked if the information was retrieved from a complete assessment of vital status or from a national death registry; for bias in measurement of the independent variable it was checked if the ascertainment of loneliness was done with a validated instrument; finally, it was evaluated if there was no bias in the selection of the reported result. In order to obtain complete information of the quality of the article, if it was part of a survey or referred to another article, the citations were consulted.

### Statistical analysis

The inter-rater agreement between the two researchers was estimated using the Kappa coefficient [25] with a confidence interval of the 95% and based on an analytical method [26]. The kappa value can be interpreted as follows: <0.20, poor; 0.21–0.40, fair; 0.41–0.60, moderate; 0.61–0.80, good; and 0.81–1.00, very good [27].

From the total articles included, those reporting a survival effect were used to conduct a meta-analysis. The effect size measures used from each article included were Hazard Ratios (HRs) and 95% CIs. Articles reporting Relative Risks (RRs) were also considered and combined with those reporting HRs. If the article did not report the confidence interval, it was calculated using the standard error. For each article included, the reported effect size (HRs or RRs) was transformed to the natural logarithms. The model used to meta-analyze the articles included was based on a random effects modeling, since it provides more conservative results than a fixed effects model [28] and assumes that each sample comes from a different population and that the effects in these populations may also differ [29]. In addition, the inverse variance weighted method was used to obtain an overall effect size and 95% CI. To evaluate if the association of loneliness with all-cause mortality is the same in women and in men, a meta-analysis was conducted by gender.

Different sensitivity analyses were carried out. Firstly, the magnitude of the effect of loneliness on all-cause mortality was assessed through a meta-analysis dividing articles with low or moderate risk of bias and articles with serious risk of bias. Secondly, a separate meta-analysis using the methodology described above was conducted over the articles reporting Odds Ratios (ORs) as an effect size measure, since ORs cannot be comparable with HRs or RRs [30]. And thirdly, in order to assess specifically the loneliness-mortality relationship independently from depression, a meta-analysis was done with the studies that controlled for depression.

The heterogeneity was evaluated by means of Cochran´s Q test at significant level of *p* < 0.10 [31] and quantified by the I^2^ statistic, considering a substantial level of heterogeneity to be 50% or more [32]. The I^2^ statistic indicates the proportion of the total variation due to that heterogeneity, while Cochran's Q measures whether the between-study variability in effect size exceeds that expected from corresponding within-study variability. Moreover, to identify potential sources of heterogeneity and characteristics related to the association of loneliness with all-cause mortality, a random effects meta-regression was employed. The characteristics considered in this analysis were: sample size (in thousands), gender of the sample (male, female or both), publication year, follow-up duration (in years), number of items of the instrument used to assess loneliness (only one item and more than one item) and quality (low or moderate risk of bias vs. serious risk of bias).

Finally, to detect publication bias, the degree of asymmetry was measured with Egger’s linear regression test [33] and Begg’s rank correlation test [34]. The former evaluates whether the association between estimated intervention effects and a measure of study size is greater than might be expected to occur by chance; and the latter assesses the correlation between test accuracy estimates and their variances. A funnel plot was done plotting the effect measure against the inverse of its standard error and included the fitted regression line from the Egger’s test for small study effects. It was considered likely publication bias if there was an asymmetric plot and *p* < 0.05. Data analysis was performed with Stata version 11 [35] using the commands *metan*, *metabias* and *metareg*.

## Results

**Fig 1:**
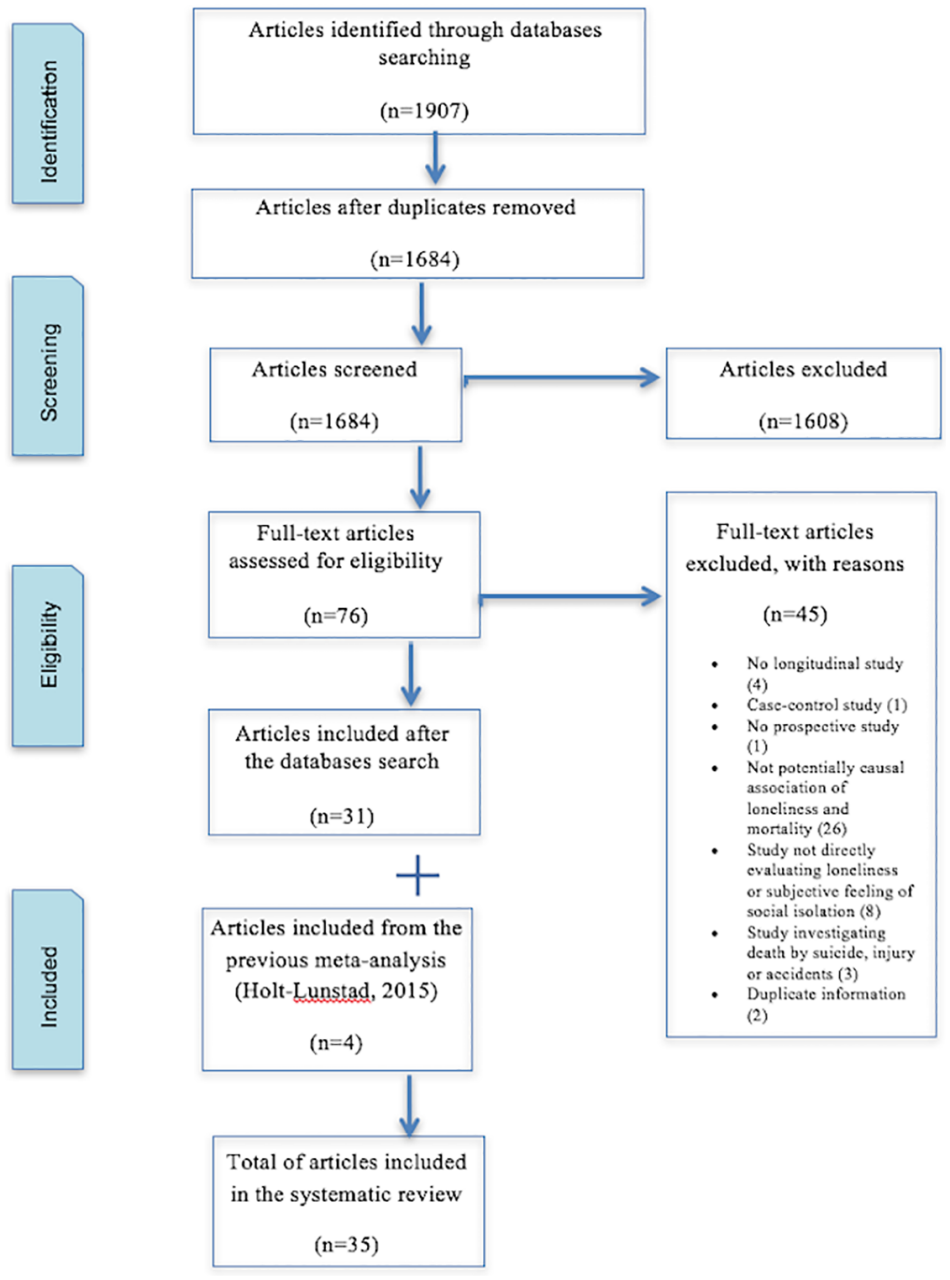
Preferred Reporting Items for Systematic Reviews (PRISMA) flow diagram.

All databases provided 1907 articles. The number of records was reduced to 1684 after duplicates were removed. After reading titles and abstracts, 1608 were excluded because they did not meet all inclusion criteria. A full-text review of 80 articles was carried out; 76 came from the databases and 4 (code: 5, 6, 16, 25) were found after examining the meta-analysis of Holt-Lunstad et al. [23]. In total, 35 articles were included in the systematic review. One of them was published in Spanish [36] and the rest were written in English. Fig 1 shows the flow diagram containing the details of the articles included and excluded.

A detailed description of the characteristics of the 35 articles included in the systematic review is reported in Table 1. From the 35 articles of the systematic review, a total of 43 studies were analyzed given that the articles that reported different effect sizes by gender (codes: 9a, 9b, 10a, 10b, 11a, 11b, 17a, 17b, 20a, 20b, 32a, 32b) or by age group (codes: 29a, 29b, 29c) were considered as different studies. For the general meta-analysis, only 24 articles were included from which 31 studies were analyzed. Twelve studies were excluded from the meta-analyses for several reasons: 5 of them reported ORs and were meta-analyzed separately (codes: 1, 5, 19, 21, 25); 6 more did not present the effect size data needed and it was not possible to obtain it even after contacting the authors (codes: 10a, 10b, 13, 15, 18, 30); and one was a meta-analysis (code: 8), whose studies are described in Table 1. Regarding the studies included in the meta-analyses, 29 were carried out in the general population (code: 3, 4, 6, 9a, 9b, 11a, 11b, 12, 14, 16, 17a, 17b, 20a, 20b, 22, 23, 24, 26, 27, 28, 29a, 29b, 29c, 31, 32a, 32b, 33, 34, 35) and 2 analyze clinical or institutionalized population (code: 2, 7).

**Table 1:** Overview of studies included in the systematic review.

STUDIES INCLUDED IN THE META-ANALYSES													
Code	First author	Year	Nation	Cohort	Follow-up	Sample age		Covariates	Mortality	Loneliness Instrument	Results	Effect Size HR (95%CI)	General or Clinical/ institutionalized
2	Drageset [11]	2013	Norway	164 f 63 m	5 Y	>65 (years)		Sx, Ag, Edu, MS, LS, CO, SI, RW, Nu, GDS.	Cancer (medical record)	Social Provisions Scale (16-items)	±	0.96 (0.90, 1.06)	CL
3	Eaker [37]	1992	USA	749 f	20 Y	45–64 (years)		Sx, Ag, S, HS, Di, BMI	Cardiovascular disease (medical record)	Are you lonely during the day? (1-item)	+	4 (1.8, 9.2)	G
4	Ellwardt [38]	2016	Netherlands	1498 f 1413 m	20 Y	55–85		Sx, Ag, De, CoD, ADL, An, Hs	All-cause (medical record)	De Jong Gierveld Loneliness Scale (11-items)	±	1.02 (0.99. 1.06)	G
6	Grand [39]	1990	France	355 f 290 m	4Y	+60 (years)		Ag	All-cause (medical record)	Do you often feel lonely? (1-item)	±	1.42 (0.81, 2.50)	G
7	Herlitz [40]	1998	Sweden	229 f 1061 m	5Y	32–86 (years)		Ag, LV, S, CHF, Di, RD, PCD, IC.	Cardiovascular disease (medical record)	"I feel lonely" (1-item from The Nottingham Health Profile)	+	1.78 (1.17, 2,71)	CL
9a	Holwerda [41]	2012	Netherlands	1509 m	10 Y	65–84 (years)		Ag, Edu, SIs, HD, Di, CD, Ca, ReD, Ar, Ep, Pa, De, CoD, ADL.	All-cause (medical record)	Do you feel lonely? (1-item)	+	1.71 (1.41, 2,07)	G
9b	Holwerda [41]	2012	Netherlands	2495 f	10 Y	65–84 (years)		Ag, Edu, SIs, HD, Di, CD, Ca, ReD, Ar, Ep, Pa, De, CoD, ADL.	All-cause (medical record)	Do you feel lonely? (1-item)	+	1.28 (1.12, 1.46)	G
11	Julsing [42]	2016	Netherlands	719 m	25 Y	64–85 (years)		Ag, Edu, S, PM, Al, BMI, CrD, DIOP, HCarD, FHSt, Chol, BPre, AnM, TDLo	All-cause (medical record)	De Jong Gierveld Loneliness Scale (11-items)	±	1.40 (0.85, 2.31)	G
12a	Jylhä [43]	1989	Finland	472 m	6,5 Y	60–89 (years)		Ag, PH, FA, DD.	All-cause (medical record)	Do you feel lonely? (1-item)	±	1.02 (0.75, 1.40)	G
12b	Jylhä [43]	1989	Finland	464 f	6,5 Y	60–89 (years)		Ag, PH, FA, DD.	All-cause (medical record)	Do you feel lonely? (1-item)	±	1.17 (0.79, 1.74)	G
13	Levy [44]	2005	USA	306 f 314 m	23 Y	50–78 (years)		Sx, Ag, MS, SRH, FH, SES.	Other cause (Respiratory mortality)	How often do you feel lonely? (1-item from Philadelphia Geriatric Center Morale Scale)	±	0.28 (0.08, 1.04)	G
15	Luo [45]	2012	USA	1253 f 848 m	6 Y	+50 (years)		Sx, Ag, Edu, MS, RFLN, SRH, Sl, PE, S, De, FL, RE, HA, HI.	All-cause (medical record)	UCLA Loneliness Scale (3-items)	±	1.07 (0.99, 1.17)	G
17	Maier [46]	1999	Germany	256 f 257 m	7 Y	70–103 (years)		Not controlled by covariates	All-cause (medical record)	UCLA Loneliness Scale (4-items)	+	1.28 (1.14, 1.44)	G
18a	Meller [36]	2004	Germany	82 m	5 Y	+85 (years)		Sx, Ag, De, Cr, Ho, Ti, An, LI, RA, CRA.	All-cause (unknown)	Geriatric Mental State (51-items)	+	1.67 (0.71, 3.87)	G
18b	Meller [36]	2004	Germany	276 f	5 Y	+85 (years)		Sx, Ag, De, Cr, Ho, Ti, An, LI, RA, CRA.	All-cause (unknown)	Geriatric Mental State (51-items)	+	1.79 (1.03, 3.09)	G
21a	Olsen [47]	1991	Denmark	715 m	16 Y	70–100 (years)		Ag, SAH, TLH, NoH5Y, SAHC, SAMH, PM, NEH, MPA, NEEVS.	Cardiovascular disease (medical record)	Do you feel lonely? (1-item)	+	1.70 (1.03, 2.81)	G
21b	Olsen [47]	1991	Denmark	1037 f	16 Y	70–100 (years)		Ag, SAH, TLH, NoH5Y, SAHC, SAMH, PM, NEH, MPA, NEEVS.	Cardiovascular disease (medical record)	Do you feel lonely? (1-item)	±	1.09 (0.79, 1.49)	G
23	Penninx [48]	1997	Netherlands	1452 f 1377 m	2,4 Y	55–85 (years)		Sx, Ag, Edu, SS, PCR, SD, PL, SRH, Al, and S.	All-cause (unknown)	De Jong Gierveld Loneliness Scale (11-items)	+	1.06 (1.00, 1.12)	G
24	Perissinotto [12]	2012	USA	953 f 651 m	6 Y	+60 (years)		Sx, Ag, Edu, RE, NWAB, Wo, LA, CO, S, Al, BMI, PE, HVP, De, ADL, UET, PM, Cl, In.	All-cause (relative record)	UCLA Loneliness Scale (3-items)	+	1.45 (1.11, 1.88)	G
25	Pitkala [49]	2004	Finland	354 f 137 m	10 Y	75, 80, 85 (years)		Sx, Ag, HS.	All-cause (medical record)	Do you suffer from loneliness? (1-item)	±	1.16 (0.99, 1.39)	G
27	Shiovitz-Ezra [50]	2010	USA	4486 f 3152 m	4 Y	+50 (years)		Sx, Ag, Edu, HS, FL, De.	All-cause (medical record)	Felt lonely much of the time over the past week (1-item from Center for Epidemiologic Studies Depression Scale)	+	1.83 (1.71, 1.87)	G
28	Stek [51]	2005	Germany	305 f 171 m	5Y	85 (years)		Sx, Ag, De, Edu, MS, Ins, S, Al, CrD.	All-cause (medical record)	Loneliness Scale of Tijhuis et al. (11-items)	±	1.30 (0.80, 1.90)	G
29	Steptoe [52]	2013	England	3547 f 2953 m	7.25 Y	+50 (years)		Sx, Ag, Edu, MS, RE, LSI, MI, Ca, Di, CHD, CLD, Ar, St, De, CES-D, We.	All-cause (medical record)	UCLA Loneliness Scale (3-items)	±	0.92 (0.78, 1.09)	G
30a	Stessman [53]	2014	Israel	145 f 196 m	20 Y	70–78 (years)		Sx, Edu, MS, PE, CP, Hy, HD, Di.	All-cause (medical record)	How often they felt lonely? (1-item)	±	1.06 (0.54, 2.10)	G
30b	Stessman [53]	2014	Israel	233 f 287 m	20 Y	78–85 (years)		Sx, Edu, MS, PE, CP, Hy, HD, Di.	All-cause (medical record)	How often they felt lonely? (1-item)	±	1.10 (0.69, 1.77)	G
30c	Stessman [53]	2014	Israel	351 f 354 m	20 Y	85–90 (years)		Sx, Edu, MS, PE, CP, Hy, HD, Di.	All-cause (medical record)	How often they felt lonely? (1-item)	±	0.84 (0.56, 1.27)	G
32	Tilvis [14]	2011	Finland	2556 f 1131 m	4.75 Y	+74 (years)		Sx, Ag, SRH.	All-cause (medical record)	Do you suffer from loneliness? (1-item)	+	1.17 (1.02, 1.33)	G
33a	Tilvis [54]	2012	Finland	1187 m	7 Y	+75 (years)		Ag, SRH, FS, DH.	All-cause (medical record)	Do you suffer from loneliness? (1-item)	±	1.17 (0.97, 1.41)	G
33b	Tilvis [54]	2012	Finland	2671 f	7 Y	+75 (years)		Ag, SRH, FS, DH.	All-cause (medical record)	Do you suffer from loneliness? (1-item)	±	1.02 (0.89, 1.17)	G
34	Tilvis [55]	2012	Finland	1678 f 812 m	4.75 Y	+75 (years)		Sx, Ag, SWL, FN, PF, ZL, NFD.	All-cause (medical record)	Do you suffer from loneliness? (1-item)	±	1.18 (0.99, 1.42)	G
35	Zhen [56]	2015	China	2164 f 925 m	3 Y	+65 (years)		Sx, Ag, Edu, MS, S, RE, UR, In, PMC	All-cause (medical record)	Sense of loneliness (1-item)	+	1.18 (1.08, 1.25)	G
**STUDIES EXCLUDED FROM THE META-ANALYSES**													
1	Cuijpers [57]	2001	Netherlands	333 f 91 m	1 Y	84.5 (mean)		Sx, Ag, YH.	All-cause (unknown)	De Jong Gierveld Loneliness Scale (12-items)	±	*1.06 (0.94, 1.19)	CL
5	Giraldi [58]	1997	Italy	95 f	6Y	-70 (years)		Not controlled by covariates	Cancer (medical record)	UCLA Loneliness Scale (20-items)	±	* 1.93 (0.82, 4.57)	CL
8	Holt-Lunstad [23]	2015	Meta-analytic review (studies included in this meta-analysis were considered as separate in our meta-analysis)										
10a	Iecovich [59]	2011	Israel	109 m	18 Y	70–88 (years)	MS, NoC, FMC, NoH, SRH, CO, FS, ES.		All-cause (medical record)	Do you feel lonely? (1-item)	DNA	DNA	G
10b	Iecovich [59]	2011	Israel	115 f	18 Y	70–88 (years)	MS, NoC, FMC, NoH, SRH, CO, FS, ES.		All-cause (medical record)	Do you feel lonely? (1-item)	DNA	DNA	G
14	Ljungquist [60]	1996	Sweden	1062 m/f	16 Y	+67 (years)	Not controlled by covariates		All-cause (medical record)	Loneliness Index (2-items)	±	DNA	G
16	Luo [13]	2014	China	7444 f 6628 m	10 Y	+65 (years)	Sx, Ag, Edu, MS, NoC, LN, UR.		All-cause (medical record)	How often the respondent feels lonely and isolated? (1-item)	+	DNA	G
19	Miller [61]	1997	USA	205 m	3 Y	37 (mean)	HS, CD4		Other cause AIDS-related mortality	UCLA Loneliness Scale (20-items)	±	DNA	CL
20	Newall [62]	2013	Canada	142 f 86 m	35 Y	77–96 (years)	Sx, Ag, MS, HS, Hap, IS.		All-cause (medical record)	De Jong Gierveld Loneliness Scale (11-items)	+	* 1.21 (1.07, 1.35)	G
22	Patterson [63]	2010	USA	3679 f 3110 m	34 Y	+21 (years)	Sx, Ag, Edu, MS, NoFR, PE, S, Sl, De, RE, In.		Cardiovascular disease (medical record)	How often they feel "very lonely or remote from other people"? (1-item)	±	* 1.03 (0.76, 1.39)	G
26	Shahtahmasebi [64]	1992	England	534 m/f	8 Y	+65 (years)	Ag		All-cause (medical record)	Self-assessed loneliness (1-item) and loneliness measure (8-items)	±	* 1.40 (0.99, 1.99)	G
31	Sugisawa [65]	1994	Japan	1197 f 1003 m	3 Y	+60 (years)	Sx, Ag, Edu, MS, SC, SP, SS, SRH, CrD, FL, Al, S.		All-cause (unknown)	Sense of loneliness (1-item)	DNA	DNA	G

**Sample**: Y = year(s); m = males; f = females; m/f = overall sample, data not available by gender.

**Covariates**: Sx (Sex); Ag (Age); Edu (Education); MS (Marital Status); LS (length of stay in nursing home); CO (comorbidity); SI (social integration); RW (reassurance of worth); Nu (nurturance); GDS (Geriatric Depression Scale); LV (left ventricular ejection fraction); S (Smoking); CHF (congestive heart failure); Di (diabetes); RD (renal dysfunction); PCD (previous cerebrovascular disease); IC (intermittent claudication); SIs (social isolation); HD (heart disease); CD (cerebrovascular disease); Ca (cancer); ReD (respiratory disease); Ar (arthritis); Ep (epilepsy); Pa (Parkinson); De (depression); CoD (cognitive decline); ADL (Activities Daily Life); NoC (number of children); FMC (frequency of meeting with children); NoH (number of people living in the same household); SRH (self-rated health); FS (functional status); SES (economic status); PH (perceived health); FA (functional ability); DD (disabling disease); FH (functional health); RFLN (relatives and friends living nearby); Sl (sleep); PE (physical exercise); FL (functional limitations); RE (race/ethnicity); HA (household assets); HI (household income); LN (living in nursing home); UR (urban/rural); Cr (crying); Ho (hopelessness); Ti (tiredness); An (anxiety); LI (loss of initiative); RA (repetition of acts); CRA (compulsive repetition of acts); Ne (neuroticism); PNP (proatrial natriuretic peptide); NYHAC (New York Heart Association Classification); HS (health status); Hap (happiness); IS (income satisfaction); SAH (self-assessment of health); TLH (time since last hospitalization); NoH5Y (number of hospitalizations over past 5 years); SAHC (self-assessment of health compared with others); SAMH (self-assessment of mental health); PM (physical mobility); NEH (nurse evaluation of health); MPA (mental and physical activity); NEEVS (nurse evaluation of expected vital status next year); NoFR (number of friends and relatives); In (income); SS (social support); PCR (personal coping resources); SD (specific diseases); PL (physical limitations); Al (alcohol use); NWAB (net worth of assets and debts); Wo (working status); LA (living arrangement); BMI (body mass index); HVP (hearing and vision problems); UET (upper extremities tasks); Cl (climbing); Ins (institutionalized); CrD (presence of chronic disease); LSI (long-standing illness); MI (mobility impairment); CHD (coronary heart disease); CLD (chronic lung disease); St (stroke); CES-D (Centre for Epidemiologic Studies Depression Scale); We (wealth); CP (chronic pain); Hy (hypertension); SC (social contacts); SP (social participation); SWL (satisfied with life); FN (feeling needed); PF (plans for future); ZL (zest for life); NFD (not feeling depressive); DH (daily help); RC (residential care); GOD (goes outdoors daily); CD4 (CD4 levels); PMC (covered by public medical service); DIOP (dispositional optimism); FHSt (family history of stroke); HCarD (history of cardiovascular disease); Chol (cholesterol); BPre (blood pressure); AnM (use of antihypertensive medication); TDLo (time-dependent loneliness).

**General or Clinical/Institutionalized**: G = general population; CL = clinical/institutionalized population; G/CL = both.

**Results**:— = protective (significant); ± = null (not significant); + = harmful (significant).

**Effect Size**: HR = hazard ratio; RR = risk ratio; 95% CI = 95% Confidence Interval;

* = effect size reported in odds ratio.

**DNA**: Data not available

**Note**: The articles that reported different effect sizes by gender or by age group were considered as different studies.

### Study characteristics and quality of the studies included in the meta-analysis

The percentage of agreement between the two independent researchers regarding whether to include or exclude each article was 98.4%, and the Kappa coefficient was 0.85 [95% CI = (0.72, 0.98)], showing a high agreement.

The characteristics of the studies included in the meta-analyses are reported in Table 2. From the 31 studies included in the meta-analysis, more than half considered both genders (61.29%), 6 analyzed only men (19.35%) and other 6 analyzed women (19.35%). The association of loneliness with mortality was evaluated in 51387 participants. A total of 12 (48.00%) studies had a follow-up longer than 10 years. Most of the studies reported all-cause mortality (80.65%) rather than a specific cause. Twenty studies (64.52%) evaluated loneliness with a single item, while eleven (35.48%) used an instrument with more than one item. Regarding the effect of loneliness on mortality, 58.06% reported null effect while 41.94% reported a harmful effect.

**Table 2:** Characteristics of the studies included in the general meta-analysis.

Characteristics	n = 31
**Gender**: n (%)	
Both	19 (61.29)
Males	6 (19.35)
Females	6 (19.35)
**Sample size**: n (mean ± SE)	
Both	39011 (2053.21 ± 2116.74)
Males	4684 (780.67 ± 507.37)
Females	7692 (1282 ± 1041.73)
**Follow-up period ≥10 Y**: n (%)	12 (48.00)
**Mortality**: n (%)	
All-cause mortality	25 (80.65)
Cardiovascular mortality	4 (12.90)
Cancer mortality	1 (3.23)
Respiratory mortality	1 (3.23)
**Loneliness**	
Studies that used a single item instrument	20 (64.52)
Studies that used instruments with several items	11 (35.48)
**Effect of loneliness on mortality**: n (%)	
Protective (significant)	0
Null (not significant)	18 (58.06)
Harmful (significant)	13 (41.94)

SE: Standard error; ≥10Y: Longer than ten years.

Table 3 describes the five items and the overall score that evaluates the quality of each article included in the meta-analysis according to the ACROBAT-NSRI tool of the Cochrane group. Only 2 articles (8.33%) were qualified with a low risk of bias, 10 articles obtained a moderate risk of bias (41.67%), and 12 presented a serious risk of bias (50%).

**Table 3:** Quality evaluation of the articles included in the meta-analysis.

Article	Bias due to confounding	Bias in selection of participants	Bias in measurement of mortality	Bias in measurement of loneliness	Bias in selection of the results	OVERALL
Drageset [11]	Moderate risk	Moderate risk	Low risk	Low risk	Low risk	**Moderate risk**
Eaker [37]	Moderate risk	Moderate risk	Serious risk	Moderate risk	Low risk	**Serious risk**
Ellwardt [38]	Moderate risk	Low risk	Low risk	Low risk	Low risk	**Moderate risk**
Grand [39]	Serious risk	Moderate risk	Serious risk	Low risk	Low risk	**Serious risk**
Herlitz [40]	Serious risk	Moderate risk	Serious risk	Low risk	Low risk	**Serious risk**
Holwerda [41]	Moderate risk	Low risk	Low risk	Low risk	Low risk	**Moderate risk**
Julsing [42]	Moderate risk	Low risk	Low risk	Low risk	Low risk	**Moderate risk**
Jylha [43]	Serious risk	Low risk	Low risk	Low risk	Moderate risk	**Serious risk**
Levy [44]	Moderate risk	Moderate risk	Low risk	Low risk	Low risk	**Moderate risk**
Luo [45]	Low risk	Low risk	Low risk	Low risk	Low risk	**Low risk**
Maier [46]	Serious risk	Low risk	Low risk	Low risk	Low risk	**Serious risk**
Meller [36]	Serious risk	Low risk	Low risk	Low risk	Low risk	**Serious risk**
Olsen [47]	Moderate risk	Low risk	Low risk	Low risk	Moderate risk	**Moderate risk**
Penninx [48]	Moderate risk	Low risk	Low risk	Low risk	Low risk	**Moderate risk**
Perissinotto [12]	Low risk	Low risk	Low risk	Low risk	Low risk	**Low risk**
Pitkala [49]	Serious risk	Low risk	Serious risk	Moderate risk	Low risk	**Serious risk**
Shiovitz-Ezra [50]	Moderate risk	Low risk	Low risk	Low risk	Low risk	**Moderate risk**
Stek [51]	Low risk	Low risk	Serious risk	Low risk	Low risk	**Serious risk**
Steptoe [52]	Moderate risk	Low risk	Low risk	Low risk	Low risk	**Moderate risk**
Stessman [53]	Moderate risk	Low risk	Low risk	Low risk	Low risk	**Moderate risk**
Tilvis [14]	Serious risk	Low risk	Low risk	Moderate risk	Low risk	**Serious risk**
Tilvis [54]	Serious risk	Low risk	Low risk	Moderate risk	Low risk	**Serious risk**
Tilvis [55]	Serious risk	Low risk	Low risk	Moderate risk	Low risk	**Serious risk**
Zhen [56]	Moderate risk	Low risk	Serious risk	Moderate risk	Low risk	**Serious risk**

### Results of the meta-analysis

**Fig 2:**
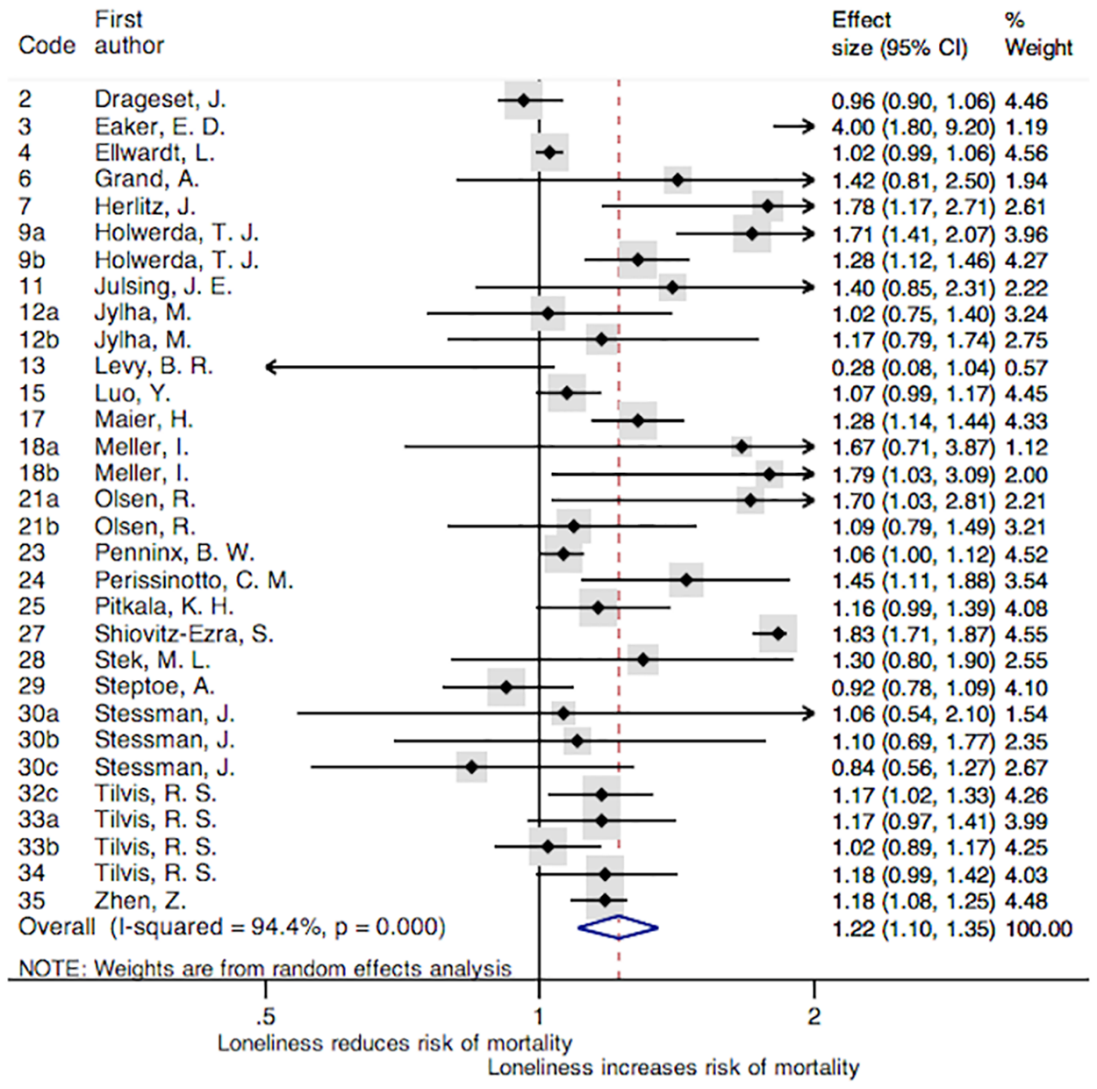
Forest plot of the studies included in the meta-analysis. **Note**: Forest plot displaying an inverse-variance weighted random-effect meta-analysis. The codes of this figure correspond to the codes of Table 1.

The association of loneliness with all-cause mortality of all the studies included in the meta-analysis is reported in Fig 2. This general meta-analysis analyzed 31 studies that came from 24 articles. The main characteristics, the effect size, the confidence interval and the percentage of weight of each study are displayed in that figure. A box has been assigned to each study; representing the weight that the study contributed to the meta-analysis. The overall combined HR were 1.22 [95% CI = (1.10, 1.35); *p* < 0.001], indicating a harmful effect of loneliness on all-cause mortality. In addition, a high heterogeneity between studies has been found (I^2^ = 94.4%), and the Cochran's Q test was significant (χ^2^(30) = 539.15, *p* < 0.001). Even excluding the six studies that only analyzed cardiovascular mortality, cancer mortality, and respiratory mortality (as can be observed in Table 2), the pooled HR associated to the remaining 25 studies was significant [HR = 1.21, 95% CI = (1.08, 1.35); *p* = 0.001] with a significant Cochran's Q test (χ^2^ (24) = 491.77, *p* < 0.001) and I^2^ = 95.1%.

A similar result was obtained when a separate meta-analysis was conducted over the five studies reporting ORs as effect size measure (code: 1, 5, 19, 21, 25, according to the notation in Table 1). The pooled OR associated with the effect of loneliness on all-cause mortality was 1.15 [95% CI = (1.03, 1.28); *p* = 0.011], indicating that loneliness was a risk factor for all-cause mortality. In this sensitivity analysis, the Cochran's Q test was not significant (χ^2^(4) = 5.68, *p* = 0.23) and the level of heterogeneity was moderate (I^2^ = 29.5%). Moreover, when the meta-analysis was restricted to the 11 studies that included depression as a covariate, loneliness was also a risk factor for mortality [HR = 1.32, 95% CI = (1.06, 1.62); *p* < 0.001], with a significant Cochran's Q test (χ^2^ (10) = 451.55, *p* < 0.001) and I^2^ = 97.8%.

**Fig 3:**
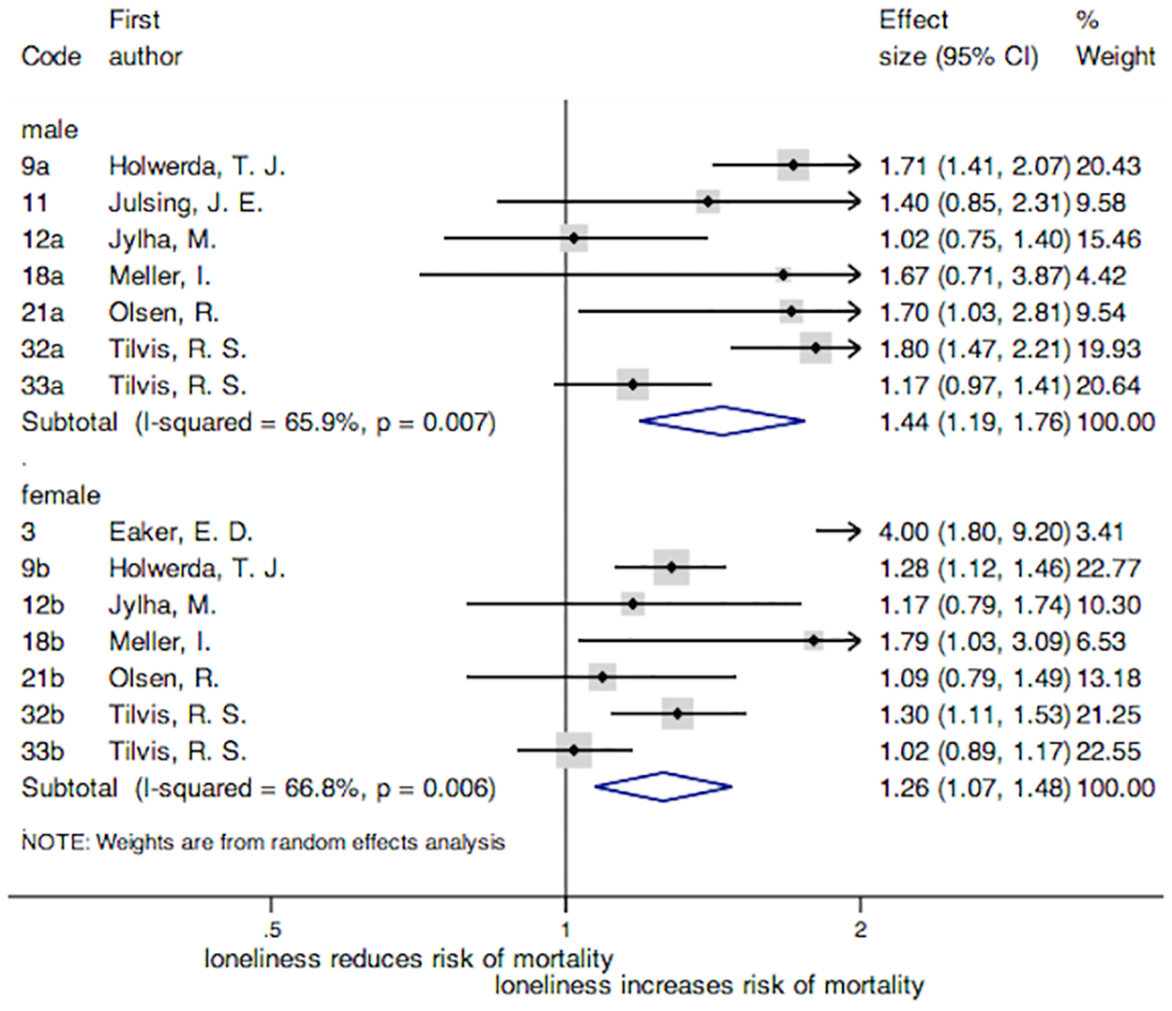
Forest plot of the studies included in the meta-analysis by gender. **Note**: Forest plot displaying an inverse-variance weighted random-effect meta-analysis. The codes of this figure correspond to the codes of Table 1.

In reference to the meta-analysis carried out by gender (Fig 3), the overall HRs were 1.26 [95% CI = (1.07, 1.48); *p* = 0.005] for women and 1.44 [95% CI = (1.19, 1.76); *p* < 0.001] for men. In both groups loneliness was a risk factor for all-cause mortality. Although significant differences in the association with mortality were not found by gender, according to the overlapping of confidence intervals, the strength of the association was slightly higher in men than in women. The heterogeneity was high in both subgroups: I^2^ = 66.8% and a significant Cochran's Q test (χ^2^(6) = 18.06, *p* = 0.006) for women, and I^2^ = 71.5% and a significant Cochran's Q test (χ^2^(6) = 17.59, *p* = 0.007) for men.

**Fig 4:**
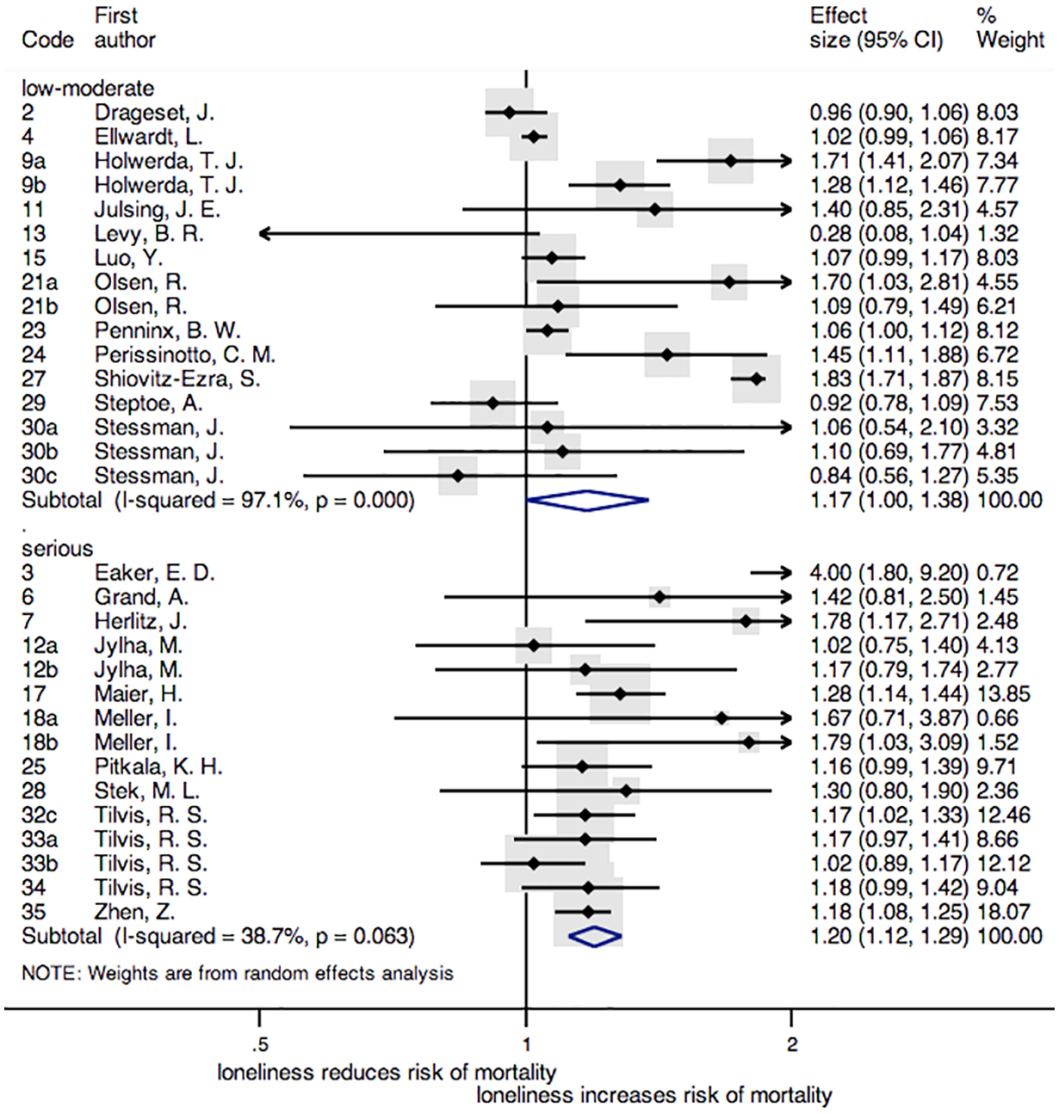
Forest plot of the studies included according to the risk of bias. **Note**: Forest plot displaying an inverse-variance weighted random-effect meta-analysis. The codes of this figure correspond to the codes of Table 1.

Considering the risk of bias of the studies, low and moderate risk versus serious risk, loneliness was also a risk factor for all-cause mortality. In the group of serious risk of bias this effect was more significant [HR = 1.20, 95% CI = (1.12, 1.29); *p* < 0.001] than in the group of low and moderate [HR = 1.17, 95% CI = (1.00, 1.38); *p* = 0.050] risk. The Cochran's Q test was χ^2^ (15) = 516.24, *p* < 0.001 for low and moderate risk and χ^2^ (14) = 22.83, *p* = 0.063 for serious risk, I^2^ values were 97.1% and 38.7% respectively (Fig 4).

In order to explore potential causes of heterogeneity and to analyze significant characteristics of the studies associated with the effect sizes obtained, a meta-regression was carried out to assess potential variables influencing the association between loneliness and all-cause mortality. However, none of the variables was found significant: the lowest *p*-value was found for follow-up [coef. = -0.02, 95% CI = (-0.04, 0.01); *p* = 0.08].

**Fig 5:**
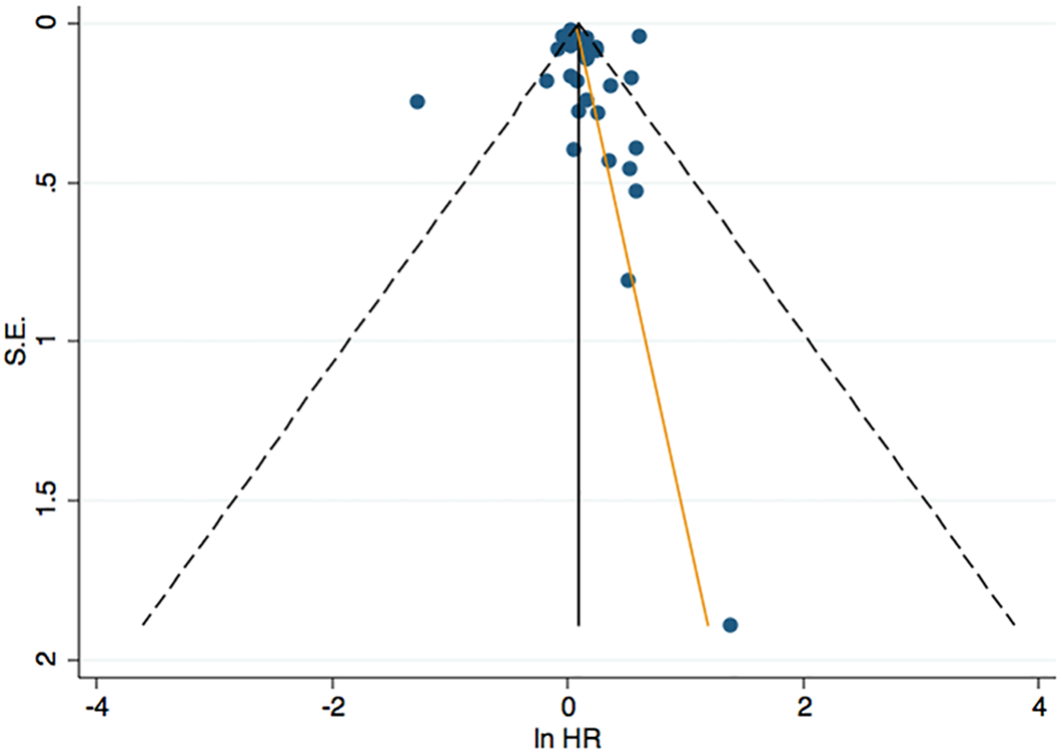
Funnel plot depicting the relationship between effect size and standard error of effect for the studies in the meta-analysis.

Based on the 31 studies included in the general analysis, potential publication bias was assessed. The publication bias is illustrated in Fig 5, where Begg's rank correlation test indicated no publication bias (*p* = 0.31), as well as Egger's linear regression: the estimated intercept for the fitted regression model was 0.60 with a standard error of 0.71, giving a *p*-value of 0.40. In Fig 5, the funnel plot appears symmetric with a distribution of the effect sizes mainly in the top and in the right side of the graph, suggesting no publication bias.

## Discussion

The overall meta-analysis shows that loneliness is a risk factor for all-cause mortality. This outcome is in line with a recently published meta-analysis that also analyzes the association of loneliness with mortality [23]. This result is consistent even after stratifying by gender, quality, and by studies that controlled for the effect of depression.

In the meta-analysis by gender, loneliness shows a tendency to be associated with all-cause mortality both in men and women. However, this effect was slightly higher in men than in women. An explanation of this difference could be that men are more reluctant to admit feelings of loneliness than women for cultural reasons [16]. Consequently, it might be that men report loneliness when its severity is high and consequently its impact is stronger. Moreover, women tend to associate loneliness with an evaluation of their overall network or relationships whereas men tend to associate this feeling with an evaluation of the relationship with their partner [19], and during the aging process the probabilities of becoming a widow increase, which might contribute to loneliness. Furthermore, widowhood has more adverse effects in men than in women and this might be because when men become widowed they have to readapt to new roles that could represent difficulties for them, like domestic tasks and assisting children [66]. The association of loneliness with health [9, 67, 68] and the fact that men generally have more negative attitudes towards care seeking [69] may also be implicated in this relationship. In addition, previous articles have shown that lonely men are more likely to suffer lower life satisfaction and higher depression, and are less resilient than lonely women [70]. In this line, some authors have suggested that the impact of social isolation on mortality might be greater in men because they experience increased inflammatory responses when they are alone than women [71]. Moreover, unhealthy lifestyles (i.e. tobacco and alcohol problems) have been associated with loneliness [72], and also more frequent in men [21], which could also explain the stronger loneliness-mortality connection in men than in women. However, the interaction of environmental and biological factors and their role needs to be further explored.

The effect of loneliness on mortality was independent from the quality of each article and the effect of depression. In spite of the frequent coexistence of loneliness with depression, particularly among older people, and the possible potential source of bias that this variable could be, it was observed that loneliness had a harmful effect on mortality in studies that controlled for depression and in studies that did not. The effect of loneliness goes beyond its associations with several health problems. Holt-Lunstad et al. [23] suggested that loneliness can be comparable with well-established risk factors for mortality. In view of the results found in this and in the previous meta-analysis, as well as in the literature that showed the harmful effect of loneliness on health [65, 73–77], it seems that it is important to consider loneliness a topic of interest for public health. There is substantial body of literature that raises the warning regarding the effects of loneliness [3, 23, 78–81].

The strengths of this work include its high sensitive search that covered all years and languages, and the inclusion of a revision of references of previous reviews and meta-analyses related to the topic of interest. Moreover, this meta-analysis updates the data regarding the association of loneliness with all-cause mortality and includes a higher number of articles and a higher number of participants compared with the previous meta-analysis. Additionally, an evaluation of the quality of each included article was done in order to analyze if the association of loneliness with all-cause mortality differs according to the quality of the studies. Furthermore, good agreement between the reviewers who did the double-check of the articles was found. To our knowledge, this is the first meta-analysis that evaluates this association of loneliness with all-cause mortality in both genders independently.

A number of limitations should be born in mind when interpreting the results. First, even though the included articles are longitudinal, causality cannot be inferred since all of them are observational. Second, only articles published in peer-reviewed journals were included, “grey literature” was excluded, which may have limited the findings. Third, the systematic review was done only in four databases (CINAHL, Pubmed, PsycINFO, and Scopus). These databases comprise a high number of articles focused in our area of interest and were chosen after consultation with an information specialist and carefully reading the descriptions of the databases. Fourth, high levels of heterogeneity, mainly in the analysis of low-moderate risk of bias studies, were obtained. Some reasons that might explain this high heterogeneity are: a high diversity of instruments used to measure loneliness, a large variety of covariates used in each study to control their effect in the association between loneliness and mortality, the wide range of publication year, the age differences analyzed in each study, and the contrast between the sample sizes. Fifth, in some cases it was not possible to obtain the necessary information to include some studies in the meta-analysis (e.g. standard deviation, confidence intervals, a measure of the effect size of loneliness, the sample size, or a comparable effect size value) even after contacting the authors. Sixth, the double-checked was done only for the 20% of the articles after removing the duplicates. Previous studies also re-inspected 20% [82–86].

Despite these limitations, some conclusions can be drawn from this article. Loneliness is a risk for all-cause mortality and this effect is slightly stronger in men than in women. The harmful of effect of loneliness on mortality was consistent across studies with different quality as well as when depression was considered as a covariate. Qualitative studies that help to understand the differential experience and the possible related factors to loneliness in men and women, and articles that use validated questionnaires for loneliness, are needed. Further studies should evaluate the association of loneliness with all-cause mortality across age, especially in the young population. Only five articles analyzed in this work had a sample younger than 50 years [37, 40, 58, 61, 63]. Besides, more articles with clinical or institutionalized population are needed since only two studies [11, 40] of those included in the meta-analysis performed analyses with this population.

Understanding the differential impact of loneliness in women and men is crucial to develop a better understanding of the nature of these feelings and approach the circumstances of the risk group. More research by gender is required to clarify and fully explore the possible association of loneliness with all-cause mortality and to suggest future recommendations in relation to prevention and treatment.

## Supporting information

S1 Dataset Dataset providing the information of all the articles included in the meta-analysis. (DTA)

S1 Text Detailed protocol followed to carry out the meta-analyses. (DOC)
