# Supplementary material for: Association of loneliness with all-cause mortality: A meta-analysis
Source: PLoS One. 2018 Jan 4;13(1):e0190033. doi: 10.1371/journal.pone.0190033 (PMC5754055; doi:10.1371/journal.pone.0190033)
Supplement: S1 Text — (DOC) [file pone.0190033.s002.doc]

**Protocol of the article “Association of Loneliness with All-cause Mortality: A Meta-Analysis”**

**Goals**

The main aim of this meta-analysis is to determine whether loneliness is associated with all-cause mortality, considering all populations (including general and clinical populations). A secondary aim is to check whether this association is the same in women and men. Additionally, this meta-analysis has been conducted with no languages and time restrictions.

**Methods**

- **Design:**

The design is a systematic literature review to identify the effects of loneliness on all-cause mortality.

- **Objects:**

The objects are the articles focusing on loneliness and mortality.

Loneliness: defined as a subjective feeling that accompanies the perception that one’s social needs are not being met by the quantity or especially the quality of one’s social relationships.

Mortality: all-cause mortality.

- **Literature search strategy:**

1. Criteria for considering articles for the review.

| - Adults older than 18 years. | Population |
| --- | --- |
| - Longitudinal observational studies. | Design |
| - Prospective cohort design. |
| - Meta-analysis. - Systematic reviews. |
| - All languages full-length publication in peer reviewed journals. | Formal Criteria |
| - Investigation of a potentially causal association of loneliness and mortality. - Investigations that measure and report loneliness quantitatively. |

1. Exclusion criteria for the articles:

| - Non-human population. - Articles investigating death by suicide, injury, or accidents because the aim was to analyze the association of loneliness with mortality through physical disease. | Population |
| --- | --- |
| - Articles that did not analyze loneliness and mortality. - Articles that did not evaluate loneliness or perceived feelings of social isolation but other constructs such as size of the network. - Articles that did not consider loneliness as an independent variable. - Articles that did not consider mortality as a dependent variable. |  |
| - Psychometric studies (development or validation of questionnaires or scales). | Design |
| - Studies of phase-I/II clinical trials. - Cross-sectional studies. |  |
| - Primary prevention studies. |  |
| - Ecologic studies. |  |
| - Case report/case series. |  |
| - Retrospective. - Case-control studies. |  |
| - Thesis. - Books or book sections. | Publication Type |

1. Keywords

The electronic search strategy was:

(("Loneliness"[Mesh]) OR Lone*[Title/Abstract]) OR Forlorn*[Title/Abstract]) OR Desol*[Title/Abstract]) OR ("Social Isolation"[Majr] OR "Feeling isolated"[Title/Abstract]) AND ("Mortality"[MESH] OR "Death"[Mesh] OR Decease*[Title/Abstract] OR Die[Title/Abstract] OR Dead[Title/Abstract] OR Remain alive[Title/Abstract] OR Remained alive[Title/Abstract] OR "Longevity"[Mesh] OR "Survival"[Mesh]) AND (Humans[Mesh]) AND (adult[MeSH]) NOT ("Cross-Sectional Studies"[Mesh]) NOT ("Books"[Mesh]) NOT ("Validation Studies" [Publication Type])).

- **Search methods for identification of articles.**

We searched the following electronic databases:

1. PUBMED (database from the United States National Library of Medicine [www.nlm.nih.gov/](http://www.nlm.nih.gov/)).
2. PSYCINFO (database of psychological literature, [www.apa.org/psycinfo](http://www.apa.org/psycinfo)).
3. Scopus (abstract and citation database of peer-reviewed literature in the fields of science, technology, medicine, social sciences, and arts and humanities <https://www.scopus.com/freelookup/form/author.uri> ).
4. CINAHL (database of full texts for nursing and allied health journal [http://web.a.ebscohost.com/ehost/search/advanced?sid=303ef526-173d-478a-bde7-1eb9fcdfadc3%40sessionmgr4001&vid=2&hid=4212](http://web.a.ebscohost.com/ehost/search/advanced?sid=303ef526-173d-478a-bde7-1eb9fcdfadc3@sessionmgr4001&vid=2&hid=4212)).

- **Data Collection Procedures.**

The data collection process consists of three subsequently steps.

First, the prospective and longitudinal articles investigating the effect of loneliness on mortality will be identified and selected. A software package for managing bibliographies will be used to eliminate duplicates. Titles and abstracts of the retrieved articles will be checked by one investigator for inclusion. Those articles that do not meet all the inclusion criteria according to the information provided in their title, abstract and keywords, will be excluded. A random sample of 20% of the articles will be double-checked independently by a second researcher. Initial disagreements between reviewers will be solved by discussion between the two reviewers. If there is disagreement, a third person will be consulted.

Second, all included articles will be fully read to confirm that they fulfilled all inclusion criteria.

Third, characteristics of the included articles will be extracted. If the articles carried out more than one analysis, the one that adjusted by more confounders and the one that reported more causes of mortality will be selected. Moreover, when multiple effect sizes were reported across different levels of loneliness, the effect that was reported as “often lonely” or “severe/chronic loneliness” will be extracted. Also, when effect sizes by different type of loneliness were reported, the emotional loneliness value was selected.

- **Statistical Analysis Plan & Quality Evaluation.**

The quality of each article will be evaluated with The Cochrane Risk of Bias Assessment Tool for Non-Randomized Studies of Interventions (ACROBAT-NSRI). Three of the seven items of this tool (three, four, and five) will be omitted given that articles of interventions are not included in the systematic review. Additionally, a new item will be included in order to evaluate the bias in the measurement of the independent variable (loneliness).

Descriptive reports of the effect of loneliness on mortality will be done according to the characteristic of each article included in the systematic review. Furthermore, some meta-analyses will be carried out. A first meta-analysis will be done with all the included articles in order to identify whether loneliness has an impact on all-cause mortality or not. A second meta-analysis will be run by gender to observe if the relationship of loneliness with all-cause mortality is the same among women and men. As sensitive analyses some meta-analyses will be done by publication bias, with the studies that report Odd Ratio, and with the studies that considered depression as a covariate. In addition a meta-regression will be carried out with the purpose of identify potential sources of heterogeneity and characteristics related to the association of loneliness with all-cause mortality.

- **Search:**

1. Search strategy for **PUBMED** (search carried out on June 23th, 2016)

| **Step** | **Concept** | **Search Statement** | **Hits** |
| --- | --- | --- | --- |
| 1 | Loneliness | Loneliness [MESH] | 2548 |
| 2 | Loneliness | Lone*[Title/Abstract] | 10118 |
| 3 | Loneliness | Forlorn*[Title/Abstract] | 18 |
| 4 | Loneliness | Desol*[Title/Abstract] | 2045 |
| 5 | Loneliness | (((("Loneliness"[Mesh]) OR Lone*[Title/Abstract]) OR Forlorn*[Title/Abstract]) OR Desol*[Title/Abstract]) | 12922 |
| 6 | Isolation | "Social Isolation"[Majr] | 6120 |
| 7 | Isolation | "feeling isolated"[Title/Abstract] | 61 |
| 8 | Isolation | ("Social Isolation"[Majr]) OR "feeling isolated"[Title/Abstract] | 6177 |
| 9 | Mortality | "mortality"[MESH] | 309332 |
| 10 | Mortality | "Death"[Mesh] | 129154 |
| 11 | Mortality | decease*[Title/Abstract] | 14206 |
| 12 | Mortality | die[Title/Abstract] | 37380 |
| 13 | Mortality | dead[Title/Abstract] | 42791 |
| 14 | Mortality | remain alive[Title/Abstract] | 2147 |
| 15 | Mortality | remained alive[Title/Abstract] | 1113 |
| 16 | Mortality | "Longevity"[Mesh] | 16947 |
| 17 | Mortality | "Survival"[Mesh] | 4284 |
| 18 | Mortality | ("Mortality"[Mesh]) OR "Death"[Mesh]) OR decease*[Title/Abstract]) OR die [Title/Abstract]) OR dead [Title/Abstract]) OR remain alive [Title/Abstract]) OR remained alive [Title/Abstract]) OR "Longevity"[Mesh]) OR "Survival"[Mesh] | 525322 |
| 19 | Combination | (((((((((((("Loneliness"[Mesh]) OR Lone*[Title/Abstract]) OR Forlorn*[Title/Abstract]) OR Desol*[Title/Abstract]) OR (("Social Isolation"[Majr]) OR "feeling isolated"[Title/Abstract])) AND ((((((((("Mortality"[Mesh]) OR "Death"[Mesh]) OR decease*[Title/Abstract]) OR die [Title/Abstract]) OR dead [Title/Abstract]) OR remain alive [Title/Abstract]) OR remained alive [Title/Abstract]) OR "Longevity"[Mesh]) OR "Survival"[Mesh])) AND "Humans"[Mesh]) AND "Adult"[Mesh]) NOT "Cross-Sectional Studies"[Mesh]) NOT "Books"[Mesh]) NOT "Validation Studies" [Publication Type]  Filters: Humans; Adult: 19+ years | 213 |

1. Search strategy for **PsycINFO** (search carried out on June 23th, 2016)

| **Step** | **Concept** | **Search Statement** | **Hits** |
| --- | --- | --- | --- |
| 1 | Loneliness | DE "loneliness" | 3409 |
| 2 | Loneliness | TI lone* OR AB lone* OR KW lone* | 9275 |
| 3 | Loneliness | TI Forlorn* OR AB Forlorn* OR KW Forlorn* | 41 |
| 4 | Loneliness | TI Desol* OR AB Desol* OR KW Desol* | 148 |
|  | Loneliness | (DE "loneliness") OR (TI lone* OR AB lone* OR KW lone* ) OR (TI Forlorn* OR AB Forlorn* OR KW Forlorn*) OR (TI Desol* OR AB Desol* OR KW Desol*) | 9531 |
| 7 | Isolation | DE "SOCIAL ISOLATION" | 6140 |
| 8 | Isolation | TI "feeling isolated" OR AB "feeling isolated" OR KW "feeling isolated" | 94 |
| 9 | Isolation | (DE "SOCIAL ISOLATION") OR (TI "feeling isolated" OR AB "feeling isolated" OR KW "feeling isolated") | 6228 |
| 10 | Mortality | TI mortality OR AB mortality OR KW mortality | 31104 |
| 11 | Mortality | DE "Death and Dying" | 26254 |
| 12 | Mortality | TI decease* OR AB decease* OR KW decease* | 3250 |
| 13 | Mortality | TI die OR AB die OR KW die | 21719 |
| 14 | Mortality | TI dead OR AB dead OR KW dead | 5225 |
| 15 | Mortality | TI remain alive OR AB remain alive OR KW remain alive | 104 |
| 16 | Mortality | TI remained alive OR AB remained alive OR KW remained alive | 55 |
| 17 | Mortality | TI longevity OR AB longevity OR KW longevity | 3999 |
| 18 | Mortality | TI survival OR AB survival OR KW survival | 31826 |
| 19 | Mortality | (TI mortality OR AB mortality OR KW mortality ) OR (DE "Death and Dying") OR (TI decease* OR AB decease* OR KW decease*) OR (TI die OR AB die OR KW die) OR (TI dead OR AB dead OR KW dead) OR (TI remain alive OR AB remain alive OR KW remain alive) OR (TI remained alive OR AB remained alive OR KW remained alive) OR (TI longevity OR AB longevity OR KW longevity) OR (TI survival OR AB survival OR KW survival) | 104628 |
| 20 | Combination | ((DE "loneliness" ) OR (TI lone* OR AB lone* OR KW lone* ) OR (TI Forlorn* OR AB Forlorn* OR KW Forlorn* ) OR (TI Desol* OR AB Desol* OR KW Desol*) ) OR ((DE "SOCIAL ISOLATION")  OR (TI "feeling isolated" OR AB "feeling isolated" OR KW "feeling isolated") ) | 15342 |
| 21 | Combination | ((DE "loneliness" ) OR (TI lone* OR AB lone* OR KW lone* ) OR (TI Forlorn* OR AB Forlorn* OR KW Forlorn* ) OR (TI Desol* OR AB Desol* OR KW Desol*) ) OR ((DE "SOCIAL ISOLATION")  OR (TI "feeling isolated" OR AB "feeling isolated" OR KW "feeling isolated") ) AND (TI mortality OR AB mortality OR KW mortality ) OR (DE "Death and Dying") OR (TI decease* OR AB decease* OR KW decease*) OR (TI die OR AB die OR KW die) OR (TI dead OR AB dead OR KW dead) OR (TI remain alive OR AB remain alive OR KW remain alive) OR (TI remained alive OR AB remained alive OR KW remained alive) OR (TI longevity OR AB longevity OR KW longevity) OR (TI survival OR AB survival OR KW survival) | 594 |
| 22 | Combination | ((DE "loneliness" ) OR (TI lone* OR AB lone* OR KW lone* ) OR (TI Forlorn* OR AB Forlorn* OR KW Forlorn* ) OR (TI Desol* OR AB Desol* OR KW Desol*) ) OR ((DE "SOCIAL ISOLATION")  OR (TI "feeling isolated" OR AB "feeling isolated" OR KW "feeling isolated") ) AND (TI mortality OR AB mortality OR KW mortality ) OR (DE "Death and Dying") OR (TI decease* OR AB decease* OR KW decease*) OR (TI die OR AB die OR KW die) OR (TI dead OR AB dead OR KW dead) OR (TI remain alive OR AB remain alive OR KW remain alive) OR (TI remained alive OR AB remained alive OR KW remained alive) OR (TI longevity OR AB longevity OR KW longevity) OR (TI survival OR AB survival OR KW survival)NOT (TI validation studies OR AB validation studies OR KW validation studies) NOT (TI cross-sectional OR AB cross-sectional OR KW cross-sectional) | 575 |
| 23 | Combination | ((DE "loneliness" ) OR (TI lone* OR AB lone* OR KW lone* ) OR (TI Forlorn* OR AB Forlorn* OR KW Forlorn* ) OR (TI Desol* OR AB Desol* OR KW Desol*) ) OR ((DE "SOCIAL ISOLATION")  OR (TI "feeling isolated" OR AB "feeling isolated" OR KW "feeling isolated") ) AND (TI mortality OR AB mortality OR KW mortality ) OR (DE "Death and Dying") OR (TI decease* OR AB decease* OR KW decease*) OR (TI die OR AB die OR KW die) OR (TI dead OR AB dead OR KW dead) OR (TI remain alive OR AB remain alive OR KW remain alive) OR (TI remained alive OR AB remained alive OR KW remained alive) OR (TI longevity OR AB longevity OR KW longevity) OR (TI survival OR AB survival OR KW survival)NOT (TI validation studies OR AB validation studies OR KW validation studies) NOT (TI cross-sectional OR AB cross-sectional OR KW cross-sectional)  **Filtros:**  Limitadores - Tipo de publicación: All Journals  Grupos de edad: Adulthood (18 yrs & older)  Grupo de población: Human | 226 |

1. **Search strategy for CINAHL (search carried out on June 23th, 2016)**

| **Step** | **Concept** | **Search Statement** | **Hits** |
| --- | --- | --- | --- |
| 1 | Loneliness | (MH "Loneliness") | 1637 |
| 2 | Loneliness | TI lone* OR AB lone* | 2580 |
| 3 | Loneliness | TI Forlorn* OR AB Forlorn* | 4 |
| 4 | Loneliness | TI Desol* OR AB Desol* | 25 |
|  | Loneliness | ((MH "Loneliness") OR (TI lone* OR AB lone*) OR (TI Forlorn* OR AB Forlorn*) OR (TI Desol* OR AB Desol*) | 3223 |
| 7 | Isolation | (MH "Social Isolation") | 4208 |
| 8 | Isolation | TI "Feeling isolated" OR AB "Feeling isolated" | 38 |
| 9 | Isolation | ((MH "Social Isolation") OR (TI "Feeling isolated" OR AB "Feeling isolated")) | 4241 |
| 10 | Mortality | (MH "Mortality") | 15223 |
| 11 | Mortality | (MH "Death") | 9750 |
| 12 | Mortality | TI decease* OR AB decease* | 1714 |
| 13 | Mortality | TI die OR AB die | 5851 |
| 14 | Mortality | TI dead OR AB dead | 2844 |
| 15 | Mortality | TI remain alive OR AB remain alive | 67 |
| 16 | Mortality | TI remained alive OR AB remained alive | 66 |
| 17 | Mortality | (MH "Longevity") | 2261 |
| 18 | Mortality | (MH "Survival") | 20188 |
| 19 | Mortality | (MH "Mortality") OR (MH "Death") OR (MH "Longevity") OR (MH "Survival") OR (TI decease* OR AB decease*) OR (TI die OR AB die) OR (TI dead OR AB dead) OR (TI remain alive OR AB remain alive) OR (TI remained alive OR AB remained alive) | 55206 |
| 20 | Combination | ((MH "Loneliness") OR (TI lone* OR AB lone*) OR (TI Forlorn* OR AB Forlorn*) OR (TI Desol* OR AB Desol*)) OR ((MH "Social Isolation") OR (TI "Feeling isolated" OR AB "Feeling isolated")) | 7117 |
| 21 | Combination | ((MH "Loneliness") OR (TI lone* OR AB lone*) OR (TI Forlorn* OR AB Forlorn*) OR (TI Desol* OR AB Desol*)) OR ((MH "Social Isolation") OR (TI "Feeling isolated" OR AB "Feeling isolated")) AND (MH "Mortality") OR (MH "Death") OR (MH "Longevity") OR (MH "Survival") OR (TI decease* OR AB decease*) OR (TI die OR AB die) OR (TI dead OR AB dead) OR (TI remain alive OR AB remain alive) OR (TI remained alive OR AB remained alive) | 229 |
| 22 | Combination | ((MH "Loneliness") OR (TI lone* OR AB lone*) OR (TI Forlorn* OR AB Forlorn*) OR (TI Desol* OR AB Desol*)) OR ((MH "Social Isolation") OR (TI "Feeling isolated" OR AB "Feeling isolated")) AND (MH "Mortality") OR (MH "Death") OR (MH "Longevity") OR (MH "Survival") OR (TI decease* OR AB decease*) OR (TI die OR AB die) OR (TI dead OR AB dead) OR (TI remain alive OR AB remain alive) OR (TI remained alive OR AB remained alive) NOT  (MH "Validation Studies") NOT (MH "Cross Sectional Studies") | 224 |
| 23 | Combination | ((MH "Loneliness") OR (TI lone* OR AB lone*) OR (TI Forlorn* OR AB Forlorn*) OR (TI Desol* OR AB Desol*)) OR ((MH "Social Isolation") OR (TI "Feeling isolated" OR AB "Feeling isolated")) AND (MH "Mortality") OR (MH "Death") OR (MH "Longevity") OR (MH "Survival") OR (TI decease* OR AB decease*) OR (TI die OR AB die) OR (TI dead OR AB dead) OR (TI remain alive OR AB remain alive) OR (TI remained alive OR AB remained alive) NOT  (MH "Validation Studies") NOT (MH "Cross Sectional Studies")  Filtros:  Limitadores - Tipo de publicación: All Journals  Especificar por SubjectAge: All adult | 121 |

1. **Search strategy for Scopus (search carried out on June 27th, 2016)**

| **Step** | **Concept** | **Search Statement** | **Hits** |
| --- | --- | --- | --- |
| 1 | Loneliness | INDEXTERMS (**LONELINESS**) | 4775 |
| 2 | Loneliness | INDEXTERMS (**LONE***) | 6378 |
| 3 | Loneliness | TITLE-ABS-KEY (**forlorn*** ) | 122 |
| 4 | Loneliness | INDEXTERMS( **desol***) | 845 |
| 5 | Loneliness | (**INDEXTERMS (LONELINESS**)) OR (**INDEXTERMS (LONE***)) OR (**TITLE-ABS-KEY (forlorn*** )) OR (**INDEXTERMS( desol***)) | 7343 |
| 6 | Isolation | INDEXTERMS( **"social isolation"** ) | 20731 |
| 7 | Isolation | TITLE-ABS-KEY ( **"Feeling isolated"** ) | 106 |
| 8 | Isolation | (**INDEXTERMS( "social isolation"** )) OR (**TITLE-ABS-KEY ( "Feeling isolated"** )) | 20819 |
| 9 | Mortality | INDEXTERMS (**mortality**) | 670350 |
| 10 | Mortality | INDEXTERMS ( **"Death"** ) | 423718 |
| 11 | Mortality | INDEXTERMS ( **decease*** ) | 169 |
| 12 | Mortality | INDEXTERMS ( **die** ) | 24411 |
| 13 | Mortality | INDEXTERMS ( **dead** ) | 18532 |
| 14 | Mortality | TITLE-ABS-KEY ( **remain alive** ) | 5745 |
| 15 | Mortality | TITLE-ABS-KEY ( **remained**  **alive** ) | 3259 |
| 16 | Mortality | INDEXTERMS( **"Longevity"**) | 26147 |
| 17 | Mortality | INDEXTERMS( **"Survival"**) | 875739 |
| 18 | Mortality | (**(((((((INDEXTERMS (mortality**)) OR (**INDEXTERMS ( "Death"** ))) OR (**INDEXTERMS ( decease*** ))) OR (**INDEXTERMS ( die** ))) OR (**INDEXTERMS ( dead** ))) OR (**TITLE-ABS-KEY ( remain alive** ))) OR (**TITLE-ABS-KEY ( remained alive** ))) OR (**INDEXTERMS( "Longevity"**))) OR (**INDEXTERMS( "Survival"**)) | 1728737 |
| 19 | Combination | ( ( ( INDEXTERMS ( **loneliness** ) )  OR  ( INDEXTERMS ( **lone*** ) )  OR  ( TITLE-ABS-KEY ( **forlorn*** ) )  OR  ( INDEXTERMS ( **desol*** ) ) )  OR  ( ( INDEXTERMS ( **"social isolation"** ) )  OR  ( TITLE-ABS-KEY ( **"Feeling isolated"** ) ) ) )  AND  ( ( ( ( ( ( ( ( ( INDEXTERMS ( **mortality** ) )  OR  ( INDEXTERMS ( **"Death"** ) ) )  OR  ( INDEXTERMS ( **decease*** ) ) )  OR  ( INDEXTERMS ( **die** ) ) )  OR  ( INDEXTERMS ( **dead** ) ) )  OR  ( TITLE-ABS-KEY ( **remain**  **alive** ) ) )  OR  ( TITLE-ABS-KEY ( **remained**  **alive** ) ) )  OR  ( INDEXTERMS ( **"Longevity"** ) ) )  OR  ( INDEXTERMS ( **"Survival"** ) ) )  AND NOT  ( INDEXTERMS ( **cross-sectional** ) )  AND NOT  ( INDEXTERMS ( **validation** ) )  AND  ( EXCLUDE ( DOCTYPE ,  **"no"** )  OR  EXCLUDE ( DOCTYPE ,  **"cp"** ) )  AND  ( EXCLUDE ( EXACTKEYWORD ,  **"Adolescent"** ) )  AND  ( EXCLUDE ( SRCTYPE ,  **"k"** ) ) | 1346 |
